# Back in the saddle: student response to remote online equine science classes

**DOI:** 10.1093/tas/txaa218

**Published:** 2020-11-21

**Authors:** Courtney Merson, Francisco Javier Navas Gonzalez, Emma Orth, Anneli Adams, Amy McLean

**Affiliations:** 1 University of California Davis, International Division of Continuing and Professional Education, Davis, CA; 2 Department of Genetics, Faculty of Veterinary Sciences, University of Córdoba, Córdoba, Spain; 3 University of California Davis, Animal Biology, Davis, CA; 4 University of California Davis, Animal Science, Davis, CA

**Keywords:** equine, remote learning, precollege, equine science

## Abstract

The COVID-19 pandemic has challenged professors and students of all disciplines to adjust quickly to remote online teaching and education platforms. In this new era of remote teaching, a greater challenge has been presented in the field of equine science; how to effectively share knowledge that is most often demonstrated by providing students access to live, in-person animal examples. Historically, students and teachers believed skill sets, which are vital for future careers in the industry (e.g., veterinarian) must be learned through hands-on experience. However, in-person methods were not available, so students were taught through the Zoom platform. Students enrolled in various levels of equine science classes were invited to complete a short voluntary questionnaire measuring their response and perception to equine courses taught in an entirely online remote setting by the same professor. One group was comprised of undergraduates majoring in the field (*n* = 44) in upper level equine science courses, Advanced Equine Reproduction Physiology and/or Equine Enterprise. These students, 41 females and 3 males, ranged in age from 20 to 25 yr, were provided a voluntary questionnaire seeking responses related to the perceived effectiveness and individual preferences of in-class lectures and in-person labs vs. remote online teaching practices. A similar questionnaire was offered on a volunteer basis to precollege students (*n* = 17). These students, female, high-school students from freshman to senior status (14–18 yr of age), were interested in equine science as a major at UC Davis in the future. This questionnaire evaluated their response to a 2-week remote synchronous online equine science course, which included multiple teaching methods, including lectures, mini labs, and full labs. Responses from both populations suggested that equine courses were perceived as effective when offered as online, remote courses. Live (synchronous) classes and labs offered on Zoom increased engagement and interaction, but students also appreciated the opportunity to access recorded materials. Students responded positively to online remote teaching and found courses to be effective for increasing their knowledge about equine science in an engaging manner, despite their continued preference for in-person instruction.

## INTRODUCTION

Spring brought a swift change across the field of education, due to varying responses to the COVID-19 pandemic from elementary schools to universities. Many responses from schools included mandated changes to pedagogy, which happened suddenly and somewhat unexpectedly for students and teachers. For example, at the University of California, Davis, all in-person classes were cancelled 9 d before the end of Winter quarter (Molinaro et al., 2020). Contextually, educators quickly adapted to innovative methods to offer classes and exams through the end of the Winter quarter, developed and planned online teaching for upcoming classes in the Spring and Summer quarters.

However, the idea of moving courses online and offering online education is nothing new(Ertmer and Nour, 2007). Since the early 2000s, most institutions have increased the number of online courses and education opportunities such as advanced degrees (Ertmer and Nour, 2007). Due to this increasing buy-in by educators shown towards online higher education, in the context of a general population of students who grew up in an era where smart phones beame ubiquitous, student adaptation to emergency remote education may have been easier than many first assumed it would be (Molinaro et al., 2020). With these student attitudes in mind, faced with the restrictions placed on in-person teaching that extended through Spring and Summer, many veterinary and animal science instructors offered online courses in lieu of traditional in-person classes. This move essentially eliminated student access to hands-on teaching activities that are heavily relied upon for creating positive student engagement in equine science courses, such as the use of instructor-curated videos depicting demonstrations, labs and live demonstrations. These videos are usually shown within synchronous online class meetings, allowing teacher and student interaction during or after viewing.

The objective of the study was to measure student responses to questionnaires completed after the conclusion of the course to learn more about student perceptions of course effectiveness. Along with student opinions about the class content and teaching methods, effectiveness was also measured through reported level of engagement in these courses that relied upon the instructional support of videos shown during class meetings. Reporting attendance (as described by Stoner and Fincham, 2012) of synchronous class sessions or viewing recorded sessions asynchronously at a later time were both considered engagement for measurement purposes. A second objective looked at the strengths or improvements to teaching methods which could be made as these courses normally rely upon in-person lectures and laboratory work to reach desired learning goals and levels of engagement through the evaluation of student answers.

That the first hypothesis in this paper evaluated whether students would rather prefer synchronous lectures, which provided the opportunity to interact in real-time with their professor during lectures and labs than the use of instructional video within synchronous classes given this help increase engagement with question and answer periods. Alternative hypothesis was that students would overall continue to prefer in-person lectures over remote teaching, regardless of the effectiveness of the teaching methods described above.

## MATERIALS AND METHODS

### Students and Questionnaires

This case study focused on students enrolled in equine science courses, which would typically be offered in person, at both the undergraduate and high-school (precollege) level. All students were asked to voluntarily share their opinions and perception of the effectiveness of equine science courses offered synchronously online. Two online questionnaires concerning the effectiveness of remote online equine science courses were designed and offered for completion to the students enrolled in one of three equine science courses. The first questionnaire was comprised of 18 questions and was created using Google Forms. This questionnaire was offered on a voluntary basis to students enrolled in one of two upper division equine science courses, Advanced Equine Reproduction Physiology and Equine Enterprise. The response options were construct-specific in hopes of avoiding response bias often seen with agree/disagree response options (Saris et al., 2010). Forty-four undergraduate students (*n* = 41 females and 3 males) completed this questionnaire. Respondents ranged in age from 20 to over 25 yr of age, and all were Junior or Senior-level students, with a majority of their majors being Animal Science with an equine option.

The second questionnaire was created specifically for completion by the precollege equine science students (*n* = 17), all high school females ranging in age from 14–18 yr. There were 17 questions in this questionnaire, which was created using Qualtrics.

Both questionnaires were designed to be completed remotely online, and were made available to students via an anonymized web link on their respective course websites. While questionnaires created in Qualtrics and Google Forms have different appearances, both can be easily accessed by students via a web link and are automatically optimized for viewing on a variety of personal devices (computers, tablets, and mobile devices). Results from both questionnaires were compiled and checked for completion, and compatible questions, which allowed for comparison between questionnaires were identified and compared. Since questionnaires were on a volunteer basis and part of the courses IRB approval was not required.

Statistical Analysis

In the context of relatively limited sample sizes and given the failure to fulfill parametric assumptions (normality and homoscedasticity, among others), results from both groups were analyzed using Bayesian inference for ANOVA. Statistical differences in the mean of the possible answers for the questions reflected in Table 1 between male and female students, and between precollege and undergraduate students.

**Table 1.** Summary of factors, questions, and possible answers

Factors	Questions	Possible answers
Gender and Student Group	How effective did you think online equine science courses were compared to in person equine science classes? Online equine science classes were:	Much less effective; less effective; the same; more effective
	Do you prefer online courses over in person lectures?	Do not prefer one over the other; slightly prefer in person lectures; slightly prefer online lectures; strongly prefer in person lectures
	Did you attend live lectures?	Always attended live lectures; mostly attended live lectures; attended live lectures about half of the time; rarely attended live lectures; and never attended live lectures
	How difficult was it for you to grasp concepts online when compared to in person classes?	Much easier; slightly easier; about the same; slightly difficult; much difficult
	How useful were the recorded lectures?	Not useful; slightly useful; very useful
	Did you prefer to attend live lectures?	Always; mostly; about half the time; sometimes; never
	When taking the course, were you in a different time zone?	I was in a different time zone for part of the duration of this course; no, I was in the same time zone for the duration of this course; yes, I was in a different time zone for the duration of this course.
	When taking the course, were you in a different country?	I was in a different time zone for part of the duration of this course; no, I was in the same time zone for the duration of this course.
	Did you find Zoom easy to use?	No; yes
	Did being asked questions in lecture help you retain information?	Being asked questions was not helpful; being asked questions was slightly helpful; being asked questions was very helpful.
	Did you prefer turning assignments in online?	Neutral; slightly prefer turning in assignments in person; slightly preferred turning in assignments online; strongly preferred turning in assignments online.
	Did you find the supplemental videos helpful to reinforce lecture content?	Not helpful; slightly helpful, very helpful
	Did the videos aid in your attention being sustained?	Not useful; slightly useful, very useful
	Did you find guest lecturers effective in this online course?	Slightly effective; very effective
	Did you prefer to see your professor or guest speakers live as they taught?	Neutral; slightly preferred to see professor/guest lecturers; strongly preferred to see professor/guest lecturers
	Was course content online easy to access?	Moderately difficult to access; moderately easy to access; very easy to access
	How effective was the professor at online teaching?	Neutral; slightly ineffective, slightly effective; very effective
	If you had the option to take your equine science course online again with this professor would you do so?	No; yes
	What you liked best about your equine science course(s) online	Everything; schedule flexibility; homework helpful to learn; interactive, international; nothing; teaching skills, very practical; nothing
	What you disliked most about your equine science course(s) online	Missed practical sessions; missed more activities; nothing; own technical problems; too easy; very long; poor video quality; missed interaction; preferred live Zoom presentations than prerecorded videos
	What is your major?	Agricultural Science; Animal Science; Animal Biology, and does not apply
	What is your specialization/favorite topic?	Animal Science; Behavior/Management; Breeding/Genetics; Companion; Dentistry; Donkey Science; Endocrinology; Equine Diseases; Equine Science; Hoof/Bomechanics; Immunology; none; Nutrition; Optometry; Other Livestock; Parasitology; Soil Science
	What is your age?	≥18, 21–30 yr old

Bayesian algorithms considered by SPSS and used in this analyses are described and publicly available in IBM SPSS Statistics Algorithms version 25.0 by IBM Corp. (IBM SPSS, Armkonk, NY IBM Corp. 2017a). The tolerance value for the numerical methods and the number of method iterations was set as a default by SPSS v25.0 (IBM SPSS Windows, Armonk, NY 2017 IBM Corp. 2017b)

The estimated effect of the factors considered in predictive models, its 95% credibility interval, and the posterior distribution statistics were computed. The significant effect of a certain factor may be detected if 0 is not contained within the credibility range.

The Bayes factor (BF) was measured to quantify the strength of the evidence against null or alternative hypothesis when comparing hypothesis pairs. Jeffreys (1961) and Lee and Wagenmakers (2013) set thresholds for BF to define the significance of evidence. The Jeffrey–Zellner–Siow (JZS) mixture of g-priors (Liang et al., 2008) was used for Bayesian inference on ANOVA. JZS prior (Rouder et al., 2012) is particularly appropriate when using ANOVA as this prior is symmetric and centered at zero in line with the predictive matching criterion as reported by Bayarri et al., 2012, hence positive and negative values of the slope parameters have a priori the same probability to occur.

## RESULTS

Both genders found online courses effective without showing a preference for synchronous online courses taught live on Zoom over the recordings of these live classes. However, females and males and precollege or undergraduate students indistinctly found it much easier to grasp concepts taught online when compared to in-person classes and found recorded sessions very useful.

Despite the usefulness of recorded class sessions, female and male students mostly preferred to attend live (synchronous) courses. Indistinctly precollege or undergraduate students slightly preferred turning in assignments online. On average, precollege and undergraduate female and male students indistinctly found being asked questions combined with viewing supplemental videos from slightly to very helpful, with these videos being from slightly to very effective when helping to sustain attention. Precollege or undergraduate females or males found guests speakers very effective, slightly preferring seeing teachers and guest speakers live on Zoom. The most valued element of online course was the interactive and international element through guest speakers, while the lowest valued aspects were related to technical problems. Precollege and undergraduate either female or male students were in the same time zone and country on average and found Zoom easy to use. On average males and female students preferred lecture topics related to donkey science, behavior, nutrition, and reproduction physiology.

According to the results, precollege and undergraduate female and male student both found content easy to access and found the professor to be very effective at online teaching, indicating they would like to repeat the course with the same professor if they had the option, or would recommend the class and teacher to their peers.

Bayesian inference for ANOVA suggested no significant differences were found for any of the questions formulated across genders, except for “Did you prefer turning assignments in online?” for which a significant difference was found between female and male respondents, with females slightly preferring to hand in assignments online, while on average males prefer to hand in assignments in person. Bayesian inference for ANOVA suggested males belonged to the Animal Biology program while females belonged to the Animal Science program on average. Female age was significantly different from males’ age with females being 21 on average while males were 26, respectively.

Bayesian inference for ANOVA suggested significant differences were found for the comparison between precollege students and undergraduate students. Specifically, undergraduate students (*P* < 0.05) slightly preferred in-person lessons while precollege students slightly preferred online lessons. When attendance was considered, significant differences were found with precollege students always attending live lectures while undergraduate students only attending half of the live lectures on average. For the attendance preference, precollege students significantly always preferred to attend live lectures while undergraduate students mostly prefer to attend live lectures. Precollege students found it (*P* < 0.05) slightly helpful being asked questions, while undergraduate students found it very helpful. Precollege students found supplemental videos slightly helpful (*P* < 0.05) while undergraduate students found them very helpful. Precollege students presented a neutral (*P* < 0.05) attitude towards seeing professor or guest speakers live as they taught, while undergraduate students reported a slight preference to see professor or guest lecturers live. On average precollege and undergraduate students differed in regards to what they disliked most due to their technical problems (with WIFI, connection, among others) while undergraduate students on average did not have anything notable to mention as a complaint or negative aspect of the course (*P* < 0.05). Undergraduate student significantly belonged to the animal science program on average. Undergraduate students (*P* < 0.05) were more specialized in equine diseases or equine science in general while precollege students’ favorites topics covered were Equine Behavior and Reproduction. Student age significantly differed, with precollege students being below the age of 18 while undergraduate students were 22–23 yr old on average.

## DISCUSSION

Educators were faced with a new and unexpected challenge in Spring of 2020; how to quickly adapt to unplanned emergency remote online teaching (Daniel, 2020; Moorhouse, 2020; Wang et al., 2020). Instructors who normally used curriculum involving hands-on labs and clinical teaching with animals had to turn to using creative innovation to teach clinical skills to students enrolled in equine science and veterinary programs (Bowen, 2020). The use of online meeting platforms became normalized for classroom use, and video platforms such as Zoom became a new household term for students and teachers (O’Brien, 2020; Bowen, 2020). In the courses examined in this case study, student responses to questionnaires have shown that both undergraduate and precollege (high school) students, have quickly and efficiently adapted to remote teaching and learning.

This quick adaptation could be linked to a generation of students whose ethos easily aligns with technology (Bowen, 2020). Perhaps due to this increased fluency with multimedia technology, incorporating various forms of technology and modes of teaching within the classroom with synchronous Zoom could be why we received positive feedback in the questionnaire. For example, the showing of videos and short clips, which had been produced or chosen by the teacher or guest speakers to demonstrate and explain key concepts associated with equine science. These teaching methods were considered especially useful for increasing student understanding of equine behavior and exercise physiology. Indeed, this practice of combining interactive instructional videos and synchronous teaching, like the method which was used in the courses examined in this study, continue to be used in multiple programs to replace hands on learning or clinical experience (Gronqvist et al., 2017; Bowen, 2020; Tan et al., 2020). From an educator’s point of view, emergency remote online teaching is similar to conducting a pilot study, or an ongoing experiment. Specifically, methods must be simultaneously reviewed and improved upon to increase student engagement, and teachers must meet the new challenge of finding teaching strategies that are appealing, effective, and improve students’ learning experience while meeting the primary goal of reaching learning outcomes (August et al., 2018; Tan et al., 2020).

Based on student’s responses to our questionnaires, combining instructional videos focused on learning objectives with dedicated question and answer sessions this method seemed to increase engagement based on student participation. This method was used in all the courses examined in this case study, and students in both populations, undergraduates and precollege, found short instructional videos developed by the professor specifically for this purpose helpful and effective. Some video segments consisted of prerecorded sessions with experts on specific topics, which were shown and supplemented by providing a question and answer period with the guest lecturer and students. Based on the questionnaire results, overall the students found the courses to still be effective in terms of meeting their expectations, which aligns with reports that such methods were successful in other didactive teaching programs (August et al., 2018). Other educators have reported successfully teaching a range of scientific courses such as chemistry or ophthalmology through the use of instructional videos with question and answer periods, which have been shown to increase engagement (Tan et al., 2020; Wang et al., 2020). In this case study, all courses taught included discussion of case studies and live demonstrations shared with students. This was deemed effective by students who took classes which met for 1.5-h blocks once a week over the course of 10 wk (the undergraduate courses) as well as classes which took place from 9 a.m. to 3 p.m. daily for 2 wk (the precollege program). The level of engagement and efficacy reported by students in these extremely varying situations is compelling as an argument to use the video accompanied with question and answer sessions as a primary method of keeping students engaged in online classes.

Students enrolled in equine courses typically want to be future veterinarians, and gaining hands-on practical experience is still considered essential for their future professions. However, other fields which usually rely upon these hands-on courses are also beginning to use instrumentation videos. For example, example in one dental hygiene course, students were shown instructional videos on various procedures and then asked open-ended questions pertaining to the information shown (August et al., 2018). A similar format was used for the courses included in this current study, and similar results showing increased engagement and perceived effectiveness of teaching was found across both groups. Such techniques are thought to be creative and innovative but essential for teaching key concepts that were once taught in a hands-on class with live horses and in-person demonstrations and practice of techniques. Thus, this methodology seems to be effective for other educators training the next generation of doctors, dentist, ophthalmologists, chemists, and veterinarians, all fields which traditionally rely on hands-on classroom experiences (August et al., 2018; Bowen, 2020; Wong et al., 2020; Tan et al., 2020; Gronqvist 2017). Similarly, the results of this study showed that most students attended live (synchronous) Zoom lectures, and although some strongly preferred that teaching method, many students also reported watching the recorded sessions at least once more to improve their understanding of various concepts and effectively review demonstrations of techniques necessary to work with live equine.

One study with veterinary students found that live stream videos, case studies, and clinical cases are helping increase students’ ability to make important clinical decisions (Bowen, 2020). In the courses involved in this study, an equine behavior and perception lab that was used in all classes helped students develop this skill. After learning the basic knowledge of equine sight, sound, and learning theory through a traditional lecture format, students were able to use this remote lab to make observations and enhance their knowledge on how and why a horse may respond in the manner it does. Similar methods using online platforms and instructional based video clips have been used to teach both veterinary and equine science students about equine behavior prior to the students engaging with live horses, so this format may be both interactive and successful for increasing the level of safety and understanding for those who may have limited equine experience (Gronqvist et al., 2017). Another question covered in our case study questionnaire asked students if they preferred to see their professor during the Zoom sessions and most replied they did prefer to see the instructor vs. having the video turned off. This could possibly replace the reactions and interactions found in the classroom and also promote learning, engagement and retention (Tan et al., 2020). The greatest challenge in this area may be the issues prompted solely due to technology issues beyond the student or teacher control, such as power outages, limited bandwidth or poor Wi-Fi connectivity. As indicated by student responses in the questionnaires, these issues negatively impacted student experience of the class. This may have exacerbated a noticeable difference that was already felt between the remote and in-person experience, though it did not affect student perception of the teacher’s efficacy or willingness to communicate with students.

Though educators in this field will continue to be challenged by certain aspects of remote teaching, the content and structure of classes remains in our control, as well as our method of delivering our message. Whether we find ourselves teaching in the physical brick and mortar classroom or in this new online classroom, the possibilities discovered in this new format should encourage all educators to reach new heights of creativity. What was initially perceived as detrimental to teaching in this field may spur innovation and the development of new teaching strategies to disseminate information in nontraditional formats, such as creating learning activities and evaluation methods that promote critical thinking and enhance student’s ability (August et al., 2018; Bowen, 2020; Tan et al., 2020). Another shift for both undergraduate courses included online assignments and exams. Most students polled preferred submitting assignments and exams online. These online exams allowed for a new test format where students were asked to respond to presented case studies and scenarios. This new format was designed to help increase the student’s critical thinking skills and provide a chance to respond to real-life equine examples which they may easily face in their career as a veterinarian. This is in contrast to previous exams offered in both undergraduate courses which asked students to respond to multiple choice or true/false questions. Precollege students, who did not receive a letter grade or complete a final exam, completed daily assignments that were turned in online on the discussion board. This method was designed to help students learn from one another and improve the students’ ability to communicate with others. Unfortunately, student perception of this aspect of the course was not explicitly addressed in the questionnaire. Overall, adapting to online emergency remote learning has tested our skills as educators to continue to find ways to communicate our message, test our teaching abilities, share, and disseminate knowledge and monitor our students learning progress through online exams and final assignments (Bowen, 2020; Moorhouse, 2020; Peachey, 2017; Tan et al., 2020; Wong et al., 2020).

## CONCLUSIONS

Based on the responses from the questionnaires one could draw the conclusion that all equine science students in college and precollege during the pandemic have found online equine science courses to be a good idea, especially when taught with instructional methods that are geared toward capturing student attention and increasing engagement meaning increasing participation from students. Based on this case study, instructors may find it helpful to teach courses synchronous, have their video camera on during lecture and create short instructional videos to supplement one taught live hands on labs. Finally, methods developed for emergency online remote learning may prove useful when moving into regularly scheduled offerings of online classes, and perhaps in certain instances could even replace hands-on experiences when in-person classes resume. Although findings suggest students will continue to prefer in-person classes, the overall positive response to these methods suggests that online equine classes can be a good idea, even as we return to traditional classes.
